# Molecular Insights Into the Natural History of Anal HSIL

**DOI:** 10.1002/jmv.70397

**Published:** 2025-05-21

**Authors:** Aude Jary, Ramon P. van der Zee, Vita Jongen, Timo J. Ter Braak, Yongsoo Kim, Chris J. L. M. Meijer, Carel J. M. van Noesel, Henry J. C. de Vries, Maarten F. Schim van der Loeff, Renske D. M. Steenbergen

**Affiliations:** ^1^ Pathology Amsterdam UMC Location VU University Amsterdam The Netherlands; ^2^ Cancer Center Amsterdam, Imaging and Biomarkers Amsterdam The Netherlands; ^3^ Internal Medicine Amsterdam UMC Location University of Amsterdam Amsterdam The Netherlands; ^4^ Amsterdam Institute for Immunology and Infectious Diseases (AII) Amsterdam The Netherlands; ^5^ Department of Infectious Diseases Public Health Service Amsterdam Amsterdam The Netherlands; ^6^ Stichting HIV Monitoring Amsterdam The Netherlands; ^7^ Pathology Amsterdam UMC Location University of Amsterdam Amsterdam The Netherlands; ^8^ Dermatology Amsterdam UMC Location University of Amsterdam Amsterdam The Netherlands

**Keywords:** AIN1, AIN2, AIN3, anal lesion progression, copy number aberrations, HSIL, LSIL, methylation markers

## Abstract

Anal squamous cell carcinoma is commonly associated with human papillomavirus (HPV) infection and preceded by low‐ and high‐grade anal lesions (LSIL; HSIL). We performed a molecular comparison on paired LSIL‐ and HSIL‐lesions collected in a longitudinal fashion to assess their relationship. Fifty biopsies from 22 men diagnosed with LSIL at baseline (T0) who developed HSIL during follow‐up (T1) were subjected to a comprehensive molecular analysis: HPV‐typing and HPV16 variant, cellular DNA methylation levels, and copy number aberrations (CNA). After histopathological revision, 23 biopsies were classified as LSIL and 27 as HSIL. Both methylation levels and CNA were significantly increased in HSIL compared to LSIL. In 15 out of 22 patients, LSIL at T0 was associated with HSIL at T1. Among them, six showed HPV‐type persistence with similar or increased methylation levels and CNA in the HSIL at follow‐up. Six patients harbored a different HPV‐type in the follow‐up biopsy, while in three patients, HPV was not detected or not‐typable in one or both lesions. A subset of HSIL preceded by LSIL displayed both HPV‐type persistence and an increase in molecular alterations, suggesting that some LSIL may progress to HSIL. In contrast, the HPV‐type switch in another subset of HSIL preceded by LSIL, may suggest an alternative pathway of anal carcinogenesis, where HSIL develop directly.

## 1 Background

Globally, over 30 000 of new anal squamous cell carcinoma (ASCC) are diagnosed each year and is most commonly attributable to human papillomavirus (HPV) infection [1]. Some populations, such as people living with HIV, men having sex with men (MSM), and women with history of HPV‐related neoplasia, have a markedly increased risk of anal cancer. Specifically, MSM living with HIV have the highest incidence rate with 85 (82−89) new ASCC cases per 100 000 person‐years [2]. Similarly to cervical cancer, ASCC is preceded by low‐ and high‐grade anal squamous intraepithelial lesions (LSIL, anal intraepithelial neoplasia grade 1 (AIN1); HSIL, AIN2−3). The prevalence of HPV increases from LSIL (from 68% to 92%) to HSIL (from 83% to 96%) reaching nearly 90% in ASCC [3, 4]. However, the natural history and the proposed progression of LSIL to HSIL, and further to ASCC is poorly understood [5]. But understanding the pathophysiology is crucial to identify better those anal lesions that will progress to HSIL or cancer and to improve their clinical management, which is particularly challenging due to difficulties in safely removing lesions and avoiding posttreatment complications. For cervical cancer, it has been suggested that cervical low‐grade lesions (LSIL; CIN1) might not always precede high‐grade lesions (HSIL; CIN2‐3), with part of cervical high‐grade lesions directly developing after a persistent HPV infection [6, 7]. Due to differences in the epithelium between the sites targeted by HPV, the oncogenesis of ASCC is not necessarily the same.

During oncogenesis, genomic aberrations, such as DNA methylation and chromosomal aberrations, contribute to the development of HPV‐induced cancer [8, 9]. Host‐genome methylation is associated with the course of HPV‐related malignancies, mainly driven by the viral oncoproteins E6 and E7 [10], and often inducing silencing of tumor suppressor genes. For this reason, DNA methylation is regarded as a well‐established biomarker for predicting cancer risk in precursor lesions related to HPV [11, 12]. In cervical samples, methylation markers have shown promising results in terms of diagnostic and prognostic value to improve cervical cancer screening and management of CIN [13]. During anal carcinogenesis, DNA methylation increases significantly with the severity of the disease and high methylation levels in HSIL are associated with progression to cancer [12, 14]. In particular, a combination of methylation markers *ZNF582* and *ASCL1* shows the highest accuracy for AIN3+ detection and provides a promising tool to identify HSIL at risk of progression to cancer and in need of treatment [14, 15]. In cervical HSIL (CIN2‐3), gains or losses at several chromosomal locations have been reported, such as gains at chromosomes arms 1pq, 3q, 7, and 20q and losses at chromosomes arms 4p, 11q, 16, and 17 [8, 16, 17, 18, 19]. Like cervical HSIL, anal HSIL show recurrent gains of 1pq, 3q, and 20q [20]. A gain of 3q26 is the most frequent event in anal HSIL and anal cancer and may be an early component of anal carcinogenesis [17, 21]. These DNA aberrations associated with HSIL progression resulting from a persistent HPV infection can provide further insight into the natural history of anal HSIL.

In this study, we aimed to assess whether LSIL may progress to HSIL by a comprehensive molecular analysis of the cellular and viral genome during the course of anal carcinogenesis from LSIL to HSIL in MSM living with HIV.

## 2 Materials and Methods

### 2.1 Patients

This longitudinal molecular study included patients from a cohort study previously described by Jongen et al [22]. Briefly, between February 2008 and November 2015, 1678 MSM living with HIV were screened for anal lesions with high‐resolution anoscopy (HRA) and biopsies were collected from suspected lesions. For participants with LSIL, a follow‐up HRA was offered after 1 year (range: 0.5−2.5 years) and a new biopsy was taken if necessary (*n* = 114). Our study focused only on patients with LSIL at baseline (T0) and who were diagnosed with HSIL during the follow‐up visit (T1) (*n* = 32). Of these, we included 23 patients in the study, in whom the T0 and T1 lesions were located with matching anatomical location (within two 12 h‐clock hours). From patients 3, 5, 17, and 21, two biopsies taken from the same time point (T0 and/or T1) were included in the analysis, referred to as 3 and 3bis, 5 and 5bis, 17 and 17bis, and 21 and 21bis, respectively.

### 2.2 Histopathological Review, DNA Isolation, and HPV Typing

Formalin‐fixed paraffin‐embedded (FFPE) tissue blocks were obtained from the archive of the Department of Pathology, Amsterdam University Medical Centers (Amsterdam UMC). For further analysis, they were cut as follows: the first and last sections for the Hematoxylin and Eosin (HE) staining to confirm the presence of the lesion, four slides for immunohistochemistry p16 ^INK4^ and Ki‐67, and 40 sections of 10 µm for DNA isolation [23]. All HE staining were reviewed by two certified pathologists experienced in AIN histopathology as previously described [23].

DNA was isolated using the QIAamp DNA FFPE tissue kit (Qiagen, Hilden, Germany). HPV detection and typing was performed as previously reported [23]; (i) the presence of HPV DNA was determined by a general primer set GP5+/GP6+ followed by hybridization with an enzyme immunoassay (EIA) allowing the detection of 13 high‐risk HPV (hrHPV) types classified as Group1 (16, 18, 31, 33, 35, 39, 45, 51, 52, 58, 59) or Group 2A (56, 68) carcinogens by the International Agency for Research on Cancer; (ii) HPV typing was performed using a microsphere bead‐based assay (Luminex xMAP, Luminax Corp, Auston, TX). The detection of low‐risk HPV (lrHPV) was processed in the same way as hrHPV, with a general primer set GP5+/GP6+ followed by hybridization of PCR products with an EIA to detect the presence of types 6, 11, and other lrHPV [24].

### 2.3 HPV16 Sanger Sequencing and Lineage Analysis

In case of HPV16 persistence, HPV16 variants were analyzed by amplification of the E6 and LCR regions followed by Sanger sequencing, as reported previously [25]. Briefly, four sets of outer primers were used to produce four PCR products of approximately 500 base‐pair (bp) length. Then, nested PCR was performed using internal primer sets leading to seven PCR products sized between 211 and 350 bp (Supporting Information S1: Table 1). Sanger sequencing was performed by Macrogen (Amsterdam, The Netherlands). The reconstruction of E6 and LCR sequences was conducted on Geneious software. First, the seven fragments of each strain were mapped on the reference genome HPV16 (NC_001526) to generate the consensus sequence. Then, multiple alignment of amplified sequences was performed with Mafft and a set of 15 published sequences available on PaVE (https://pave.niaid.nih.gov/explore/variants/variant_genomes) and the GenBank database, and including different lineage/sublineage of HPV16. Finally, phylogenetic analysis was performed using PhyML, the maximum likelihood method (best model: GTR + G), and 1000 bootstrap resampling.

### 2.4 DNA Methylation Analysis Using Multiplex Quantitative Methylation‐Specific PCR (qMSP)

After bisulfite conversion of DNA using the EZ DNA Methylation kit (Zymo Research, Orange, CA, USA), DNA‐methylation analysis was performed using two qMSP, as previously reported [12]. Each qMSP targets three methylation markers (*ASCL1–LHX8–ZNF582* and *SST–WDR17–ZIC1*) and one reference gene, the ß‐actin (*ACTB*). A cycle threshold < 32 for ACTB indicated sufficient DNA and adequate bisulfite conversion. Methylation values of the targets were normalized using the comparative *C*_q_ method; ΔΔ*C*_q_ ratios were computed by comparing the target *C*_q_ values with the *C*_q_ values of *ACTB* and of the internal quality control calibrator ($2^{-\Delta \Delta C_{q}}$ × 100) [26].

### 2.5 DNA Copy Number Analysis Using modified Fast Aneuploidy Screening Test‐Sequencing System (mFAST‐SeqS)

We used the mFAST‐SeqS which targets specifically the long interspersed nucleotide element‐1 (LINE‐1) retrotransposons in the human genome, to identify copy number aberrations (CNA) in biopsies [27, 28]. Briefly, 10 ng of DNA was amplified for a first PCR targeting LINE‐1 and then a second one to add specific barcodes to identify each sample before pooling and sequencing (Supporting Information S1: Tables 2, 3, and 4) performed on iSeq. 100 system (Illumina), 150 bp single reads. After trimming of the reads using Trimmomatic, reads were mapped to the human genome hg19 using BWA and count of mapped reads using an in‐house script. After normalization of read counts on total reads per sample, calculation of arm‐chromosome specific *z*‐score, and genome‐wide *z*‐score were performed by comparison with healthy anal controls [27, 28]. To determine the presence of chromosomal gains or losses in a sample, a predetermined threshold of 4 was used for calling [28].

### 2.6 Statistical Analysis

Continuous variables were expressed as median and interquartile [IQR] and categorical variables as number and percentage (*n*, %). The nonparametric Wilcoxon signed‐rank test was performed to compare methylation levels between histological categories. Results of methylation levels and chromosomal aberrations between paired biopsies were compared manually. For statistical analysis and figures, we used the R software (v4.3.2) and packages *gt*, *gtsummary,* and *ggplot2*. *p* < 0.05 was considered statistically significant.

## 3 Results

**Figure 1:**
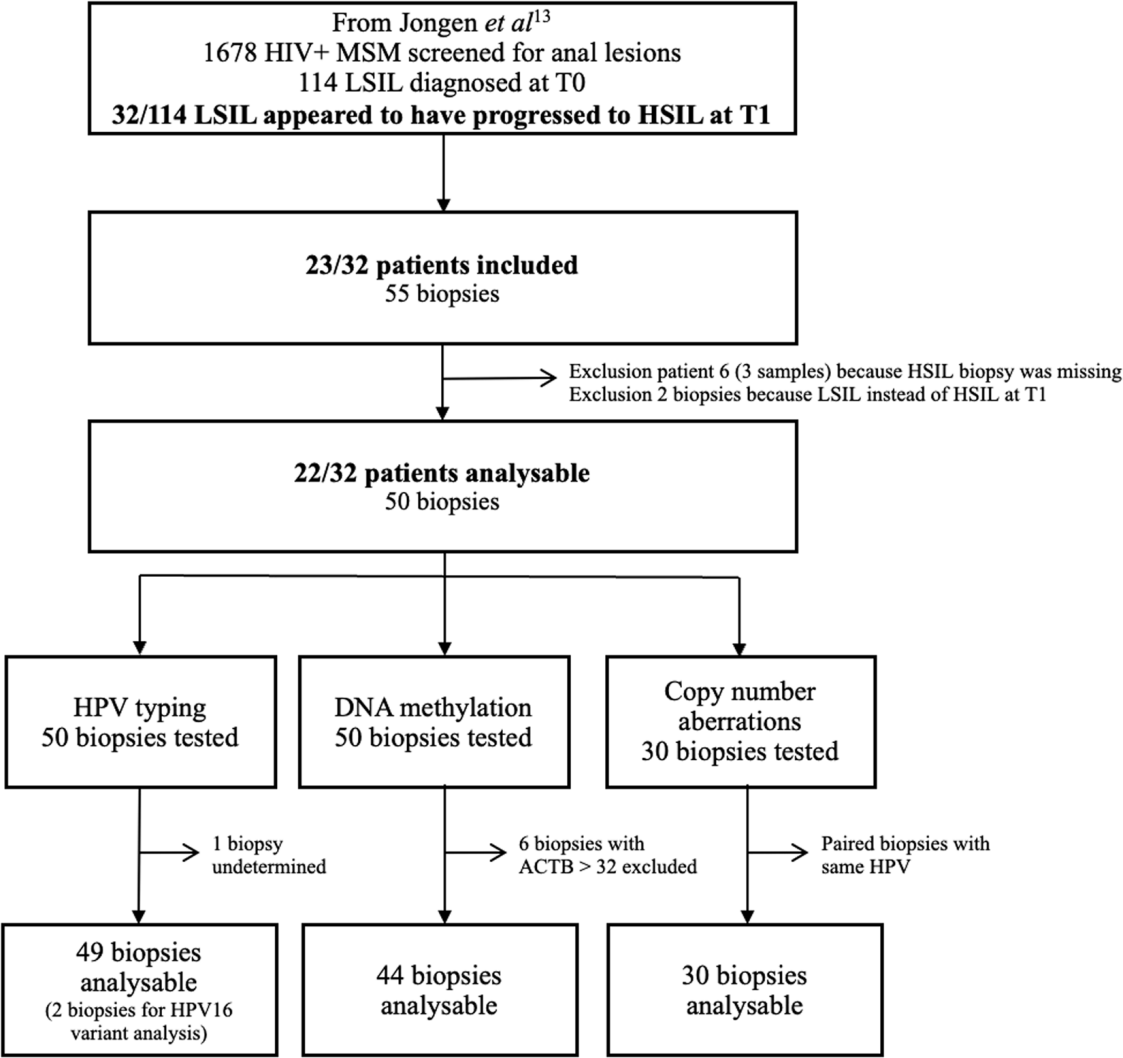
Flowchart of the study.

Between 2008 and 2015, 1678 MSM with HIV underwent initial HRA, of whom 114 patients had LSIL at baseline (T0) and returned for HRA at 1 year follow‐up (T1) [22]. Thirty‐two patients (28%) with LSIL at baseline showed progression to HSIL at the follow‐up visit, of whom 23 (72%) were included in this study based on availability of biopsy material. None of them has received the HPV‐vaccination. Of these, a total of 55 biopsies were initially collected from 16 patients with two paired biopsies (one lesion at T0 and one lesion at T1), five patients with three paired biopsies (two lesions at T0 and one lesion at T1), and two patients with four paired biopsies (four lesions at T0 and two lesions at T1). The median [IQR] time between LSIL and HSIL sampling was 13.2 months [12.2−15.7]. One patient was excluded because the HSIL sample of T1 was missing, resulting in exclusion of two biopsies at T0 and one biopsy at T1. From another two patients, of whom each had two biopsies at T1, two LSIL were also excluded. In total, 50 biopsies derived from 22 patients were used for further analysis and issued (Figure 1).

### 3.1 HPV‐Typing, DNA Methylation, and Chromosomal Aberrations in the Total Study Population

After histopathological reviewing of the tissue sections that were newly cut for the purpose of this study, three lesions were classified as no dysplasia (all at T0), 20 as LSIL/AIN1 (*n* = 17 from T0 and *n* = 3 from T1), and 27 lesions as HSIL (AIN2, *n* = 23 and AIN3, *n* = 4; *n* = 7 from T0 and *n* = 20 from T1). Most biopsies were positive for HPV (92%). The prevalence of hrHPV was 33%, 30%, and 70% in the no dysplasia, LSIL, and HSIL groups, respectively. Multiple HPV types were found in 43% of HPV‐positive biopsies. HPV16 was the most prevalent type (*n* = 8), followed by HPV33 (*n* = 5), HPV51 (*n* = 5), HPV58 (*n* = 3), and HPV59 (*n* = 3). The prevalence of lrHPV was also high with 66% overall positivity and 85% positivity in LSIL. HPV genotyping per lesion is shown in Supporting Information S2: Table 1.

**Figure 2:**
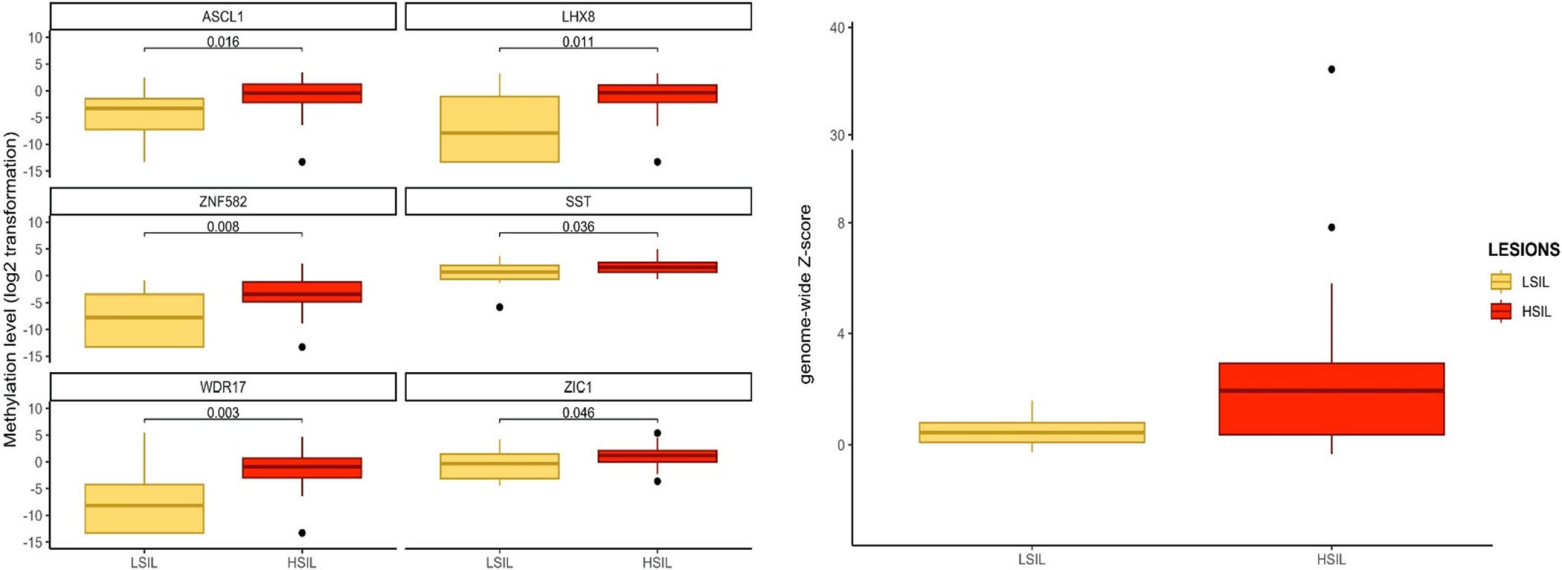
Methylation levels (A) and genome‐wide *Z*‐score (B) from anal LSIL and HSIL‐lesions collected in MSM living with HIV. No dysplasia (*n* = 3) is grouped with LSIL. HSIL, high‐grade squamous intraepithelial lesion; LSIL, low‐grade squamous intraepithelial lesion.

DNA methylation analysis was performed on 44 biopsies, excluding six biopsies because of low DNA quality (ACTB > 32; three LSIL and three HSIL). Methylation levels of all six markers were significantly increased in HSIL compared to LSIL/no dysplasia (Figure 2A). More specifically, *ZNF582* and *WDR17* methylation markers showed the largest differences in the distribution of methylation levels between LSIL/no dysplasia and HSIL (ZNF582 median [IQR]: −7.8 [−13.3, −3.5] vs. −3.4 [−4.8, −1.2], *p* = 0.008 and WDR17 median [IQR]: −8.2 [−13.3, −4.3] vs. −0.9 [−3.0, 0.7], *p *= 0.003). Median [IQR] methylation levels for each marker and in each lesion are shown in Supporting Information S2: Table 2.

**Figure 3:**
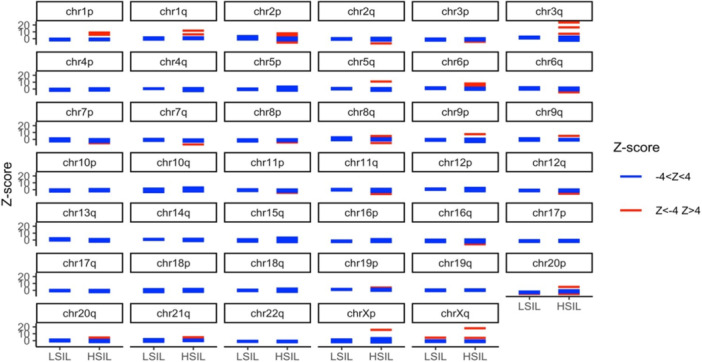
Chromosome arm‐specific *Z*‐score from anal LSIL (*n* = 11) and HSIL (*n* = 19) lesions collected in MSM living with HIV. Each line corresponds to one lesion, in blue those with a normal chromosome arm‐specific *Z*‐score and in red those with abnormal chromosome arm‐specific *Z*‐score corresponding to a gain (*z*‐score > 4) or a loss (*z*‐score < −4). No dysplasia (*n* = 3) is grouped with LSIL. HSIL, high‐grade squamous intraepithelial lesion; LSIL, low‐grade squamous intraepithelial lesion.

Chromosomal aberrations were determined when in paired T0 and T1 biopsies, the same HPV types were found, resulting in 30 biopsies being analyzed by mFAST‐SeqS. A median [IQR] of 149 587 [121 837–181 349] reads per sample was obtained and all 30 samples had enough reads for the copy number analysis. The genome‐wide *z*‐score increased from median [IQR] 0.4 [0.1−0.8] in the LSIL/no dysplasia group to median [IQR] 1.9 [0.4−2.9] in HSIL (Figure 2B). Using a previously defined *z*‐score threshold of 4 [28], four out of 19 HSIL (two AIN2 and two AIN3) scored positive for CNA, while none of the LSIL/no dysplasia biopsies displayed CNA. At the chromosomal arm level, the most frequent aberrations found in the HSIL group were gains of 6p (4/19), 2p (3/19), 3q (3/19), 1p (2/19), 1q (2/19), and Xq (2/19) (Figure 3 and Supporting Information S2: Table 3). No chromosomal arm changes were seen in LSIL and no dysplasia.

### 3.2 HPV‐Typing, DNA Methylation, and Chromosomal Aberrations in Paired Biopsies

Of the 22 patients, 15 patients (17 paired biopsies) had LSIL at baseline (T0) and HSIL during follow‐up (T1); the seven remaining patients (nine paired biopsies) had LSIL (*n* = 2) or HSIL (*n* = 7) at both sampling times.

Among the 15 patients with LSIL at T0 and HSIL at T1, 4/15 patients had lrHPV‐type persistence, 2/15 patients had hrHPV‐type persistence, and 8/15 patients harbored different HPV types in LSIL and HSIL. In one patient HPV was detected at T0 and T1, but at both time points the HPV‐type was undetermined (patient 11, data not shown).

**Figure 4:**
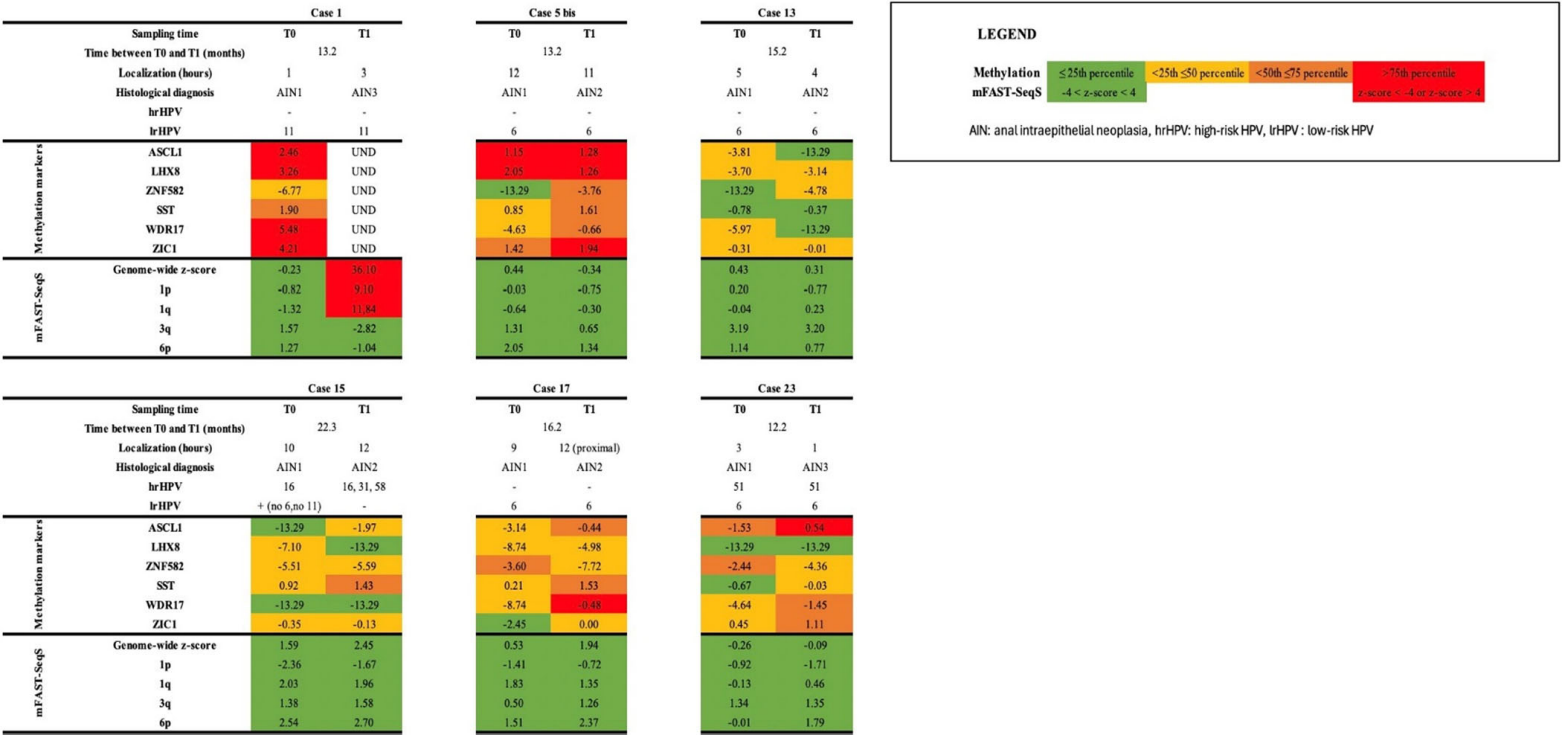
Histopathology, methylation markers, and chromosomal aberrations in patients who progressed from anal LSIL to HSIL with HPV‐persistence. Bis indicates a second biopsy form same time point. HSIL, high‐grade squamous intraepithelial lesion = AIN2 and AIN3; LSIL, low‐grade squamous intraepithelial lesion = AIN1.

Paired biopsies with HPV‐type persistence (*n* = 6 patients; *n* = 12 biopsies) showed similar or an increase of methylation levels of the six methylation markers between LSIL and HSIL, while most biopsies showed no CNA at both T0 and T1 (Figure 4). Only patient 1, with HPV11 persistence, showed a strong increase in genome‐wide *z*‐score in the HSIL biopsy (from −0.23 at T0 to 36.10 at T1), associated with gains of 1p and 1q. Unfortunately, methylation could not be determined in the HSIL (T1) biopsy for this patient. Patient 15, showing HPV16 persistence, demonstrated an increase in *ASCL1* and *SST* methylation in the HSIL (T1) biopsy, while no CNAs were seen in both LSIL and HSIL lesions. Patient 23, with persistence of HPV51, showed increased methylation of five out of six markers in the HSIL (T1) biopsy, but no CNA in both LSIL and HSIL.

Most biopsies with different HPV‐types harbored an increase of methylation levels from LSIL to HSIL (Supporting Information S2: Figure 1).

**Figure 5:**
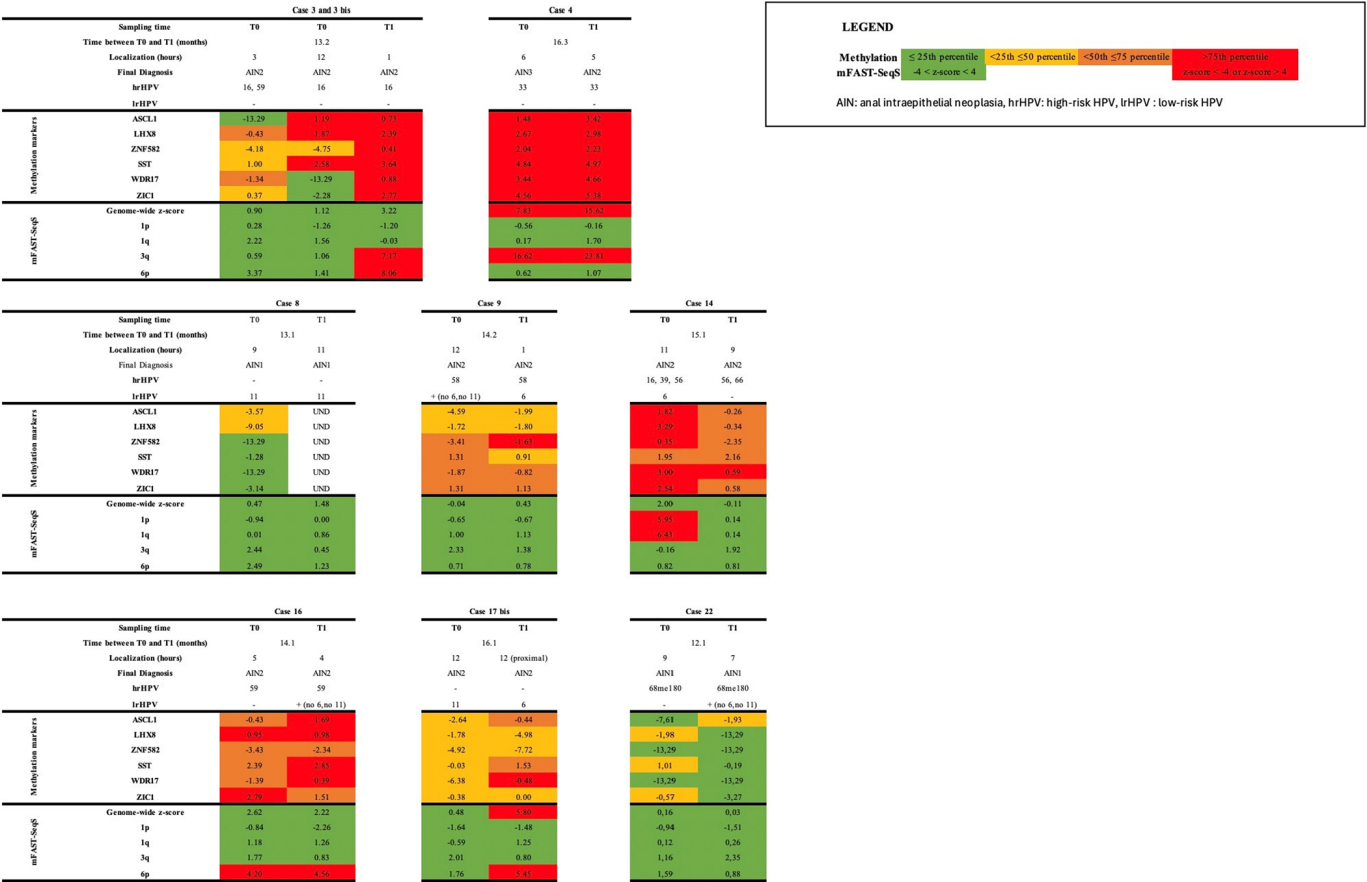
Histopathology, methylation markers, and chromosomal aberrations in patients who had a persistence of anal LSIL or HSIL lesions. Bis indicates a second biopsy form same time point. HSIL, high‐grade squamous intraepithelial lesion = AIN2 and AIN3; LSIL, low‐grade squamous intraepithelial lesion = AIN1.

Among patients with persistent lesions (same histology at T0 and T1; *n* = 8), HSIL persistence occurred in six patients and was mainly associated with hrHPV infection (*n* = 5). In these patients, a similarly high or an increase in methylation marker levels was seen in most of the biopsies at T1. CNA were less frequent, with an increased genome‐wide *z*‐score or gains of chromosomal arms, mostly cooccuring with high methylation levels (Figure 5). In patient 14, methylation levels and CNA decreased between the two‐sampling times, which was associated with the disappearance of HPV16 at T1. LSIL persistence occurred in two patients and was associated with lrHPV (HPV11) infection (patient 8) and with probable hrHPV infection (HPV68, patient 22). In all four biopsies, no/low methylation and no CNA were found.

HPV16 variant analysis was performed in case of HPV16 persistence and HSIL diagnosis, which was only seen for patients 3 and 15. Unfortunately, HPV16 variant analysis was not successful for patient 15. In patient 3, all three biopsies, two from T0 and one from T1, harbored the same HPV variant, corresponding to the sublineage A2 (Supporting Information S2: Figure 2).

## 4 Discussion

In this longitudinal study, we performed a comprehensive molecular analysis of viral and cellular DNA, in a longitudinal series of paired LSIL and HSIL lesions obtained from MSM living with HIV participating in anal cancer screening. When comparing LSIL and HSIL lesions, we observed an increase in the DNA methylation of cellular genes and chromosomal aberrations. From an initial series of 22 patients included in the study, there were 15 patients in which a LSIL was found at baseline and an HSIL at follow‐up, with six patients showing persistence of the same HPV‐type. In a subset of these cases, sequential increase of LSIL to HSIL was characterized by an increase in DNA methylation and to a lesser extent an increase of chromosomal aberrations. Persistence of HSIL was also associated with hrHPV presence and increased DNA methylation, whereas persistence of LSIL was associated with lrHPV without changes in the cellular DNA.

Analysis of this longitudinal paired‐biopsies series confirmed that methylation levels increase from LSIL to HSIL, in line with our previous findings [12, 14, 15]. We recently validated that particularly the marker combinations *ASCL1* and *ZNF582*, either or not combined with *SST*, had a very good performance for AIN3 and anal cancer detection in MSM living with HIV [12, 14, 15] and a similarly good performance to detect AIN3 and cancer in HIV‐negative MSM [29].

The increase in methylation level in lesions which progressed from LSIL to HSIL is suggested to be driven by the HPV E6 and E7 oncoproteins which upregulate the activity of the human DNA methyltransferases (DNMTs), specifically DNMT1a and DNMT3b thereby promoting tumor suppressor gene silencing by methylation of their promoter regions [30, 31]. The viral oncoproteins can also impact cellular genome integrity resulting in genomic instability associated with gains and losses of specific chromosomal regions as well as entire chromosomal arms. In contrast to the frequent increase in methylation levels, CNA determined by the mFAST‐seqS approach, were only found in a smaller subset of HSIL lesions. The most frequent CNA identified were gains of 1p, 1q, and 3q, as has also been reported before on HPV‐induced lesions from different anogenital regions [8]. Present longitudinal findings provide further proof for these events being involved in the course of anal carcinogenesis. Our data indicate that chromosomal aberrations are detectable at a later stage than DNA methylation of specific genes. Nonetheless, we cannot exclude that detection of CNA is masked by the presence of normal cells, as we did not enrich for dysplastic cells by microdissection of the lesions. Moreover, while the presence of the lesion in the sections used for DNA analysis was confirmed by histological assessment of the first and last section (sandwich method), there were a few cases in which the HE staining of the last section showed that the lesion was almost gone upon cutting the blocks

Importantly, we found more lrHPV persistence than hrHPV persistence among LSIL lesions that progressed to HSIL, which was unexpected. More specifically, in patient 1, who progressed from LSIL to HSIL with a persistence of HPV11, a high increase in CNA was found. In the literature, some studies also reported cases of HSIL and anal cancer related to lrHPV, mainly HPV6 and HPV11, in individuals living with HIV, suggesting the potential for progressive disease in HSIL lesions caused by lrHPV types, especially in immunocompromised individuals [32, 33]. Further explorations on the physiopathology of anal cancer are required to better understand the role of the lrHPV types in the persistence and progression of anal lesions.

In 9 out of 15 patients with a sequential increase of LSIL to HSIL, different HPV‐types were found in the paired‐biopsies. These cases often had multiple HPV‐infections at T0 and T1 without persistence of any of the HPV‐types, suggesting that in some cases, HSIL might have developed directly from a healthy epithelium infected with HPV. A study on progression of LSIL to HSIL found that 70% of HPV16/18 positive LSIL progressed to HSIL which was significantly higher than seen for non‐HPV16/18 positive or hrHPV negative LSIL (67% vs. 25% and 7%, respectively) [34]. In our study, only 4 out of the 27 samples tested at T0 were HPV16/18 positive, of which only one was associated with a sequential increase of LSIL to HSIL (HPV16, patient 15). Unfortunately, the HPV16 variant could not be determined in that patient to confirm the association between HPV16 persistence and lesion progression.

It is important to note that among the 22 patients initially included, seven patients were found to have persistent lesions instead of progressive lesions after revision of the slides. This observation highlights two major points: (i) as we analyzed another part of the FFPE tissue block, we cannot rule out that the initial lesion might have been cut out, resulting in four original HSIL biopsies (T1) being classified as LSIL after revision and (ii) the difficulty in AIN2 diagnosis. During revision, all the slides were stained for p16 and Ki67 resulting in three LSIL being reclassified as HSIL (AIN2).

A strength of our work is that we collected a rather unique longitudinal series of paired biopsies from MSM living with HIV, which were subjected to various molecular analysis including viral and cellular parameters. Limitations of our study are the low sample size, with a few samples being of insufficient quality or quantity for all molecular analyses. Moreover, we did not perform microdissection on the lesions, so dysplastic cells might have been diluted in our analysis, particularly hampering the identification of CNA.

In conclusion, we observed both lrHPV‐ and hrHPV‐type persistence during follow‐up in one‐third of cases, with similar or elevated levels of DNA methylation and CNA in the HSIL, suggesting that LSIL can progress to HSIL. In the remaining cases, different HPV types were found in the HSIL at follow‐up, which suggests that HSIL might develop directly without a preceding LSIL. This strengthens the evidence that anal carcinogenesis may follow two routes.

## Author Contributions

Study design: Renske D. M. Steenbergen, Ramon P. van der Zee, Maarten F. Schim van der Loeff, and Vita Jongen. Data collection: Aude Jary and Ramon P. van der Zee. Data management: Aude Jary, Ramon P. van der Zee, and Yongsoo Kim. Laboratory experiments: Aude Jary and Timo J. Ter Braak. Statistical analysis: Aude Jary. Data interpretation: Aude Jary and Renske D. M. Steenbergen. Writing first draft of manuscript: Aude Jary. All authors were involved in writing the manuscript and gave final approval of the submitted and published version of the manuscript.

## Ethics Statement

The study (reference W15_047 15.0058 and 18/341) was approved by the Medical Ethics Review Committee of the Academic Medical Center, Amsterdam.

## Conflicts of Interest

R.D.M.S. and C.J.L.M.M. are minority shareholders of Self‐screen B.V., a spin‐off company of VUmc; Self‐screen B.V. develops, manufactures and licenses high‐risk HPV and methylation marker assays for cervical cancer screening and holds patents on these tests. R.D.M.S. declares consultancy fee from Astra Zeneca. C.J.L.M.M. is part‐time CEO of Self‐Screen and served occasionally on the scientific advisory board and/or the speakers bureau of Qiagen. M.S.V.D.L. served on the Advisory Board of NovoSanis and his institution receives funding from GSK for an investigator‐initiated study. The other authors declare no conflicts of interest.

## Supporting information

Supplementary Material.

SupplementaryResults.

## Data Availability

The data underlying the analysis of our study are in anonymized form available from the corresponding author upon reasonable request and following the datat protection regulations.
