# Supplementary material for: Molecular Insights Into the Natural History of Anal HSIL
Source: J Med Virol. 2025 May 21;97(5):e70397. doi: 10.1002/jmv.70397 (PMC12096055; doi:10.1002/jmv.70397)
Supplement: Supplementary file 1 — Supplementary Material. [file JMV-97-e70397-s002.docx]

**SUPPLEMENTARY MATERIALS**

**Amplification of HPV16 E6 and LCR viral genome regions**

**Table 1**: Primers sequences

| **Names** | **Sequences (5′ - 3′)** | **Nucleotide positions** | **Product size (bp)** |
| --- | --- | --- | --- |
| 16E6-1F-outer | ttgaaccgaaaccggttagt | 46–65 | 393 |
| 16E6-2R-outer | ggacacagtggcttttgaca | 419–438 |  |
| 16E6-1F-inner | ttgaaccgaaaccggttagt | 46–65 | 211 |
| 16E6-1R-inner | gcataaatcccgaaaagcaa | 237–256 |  |
| 16E6-2F-inner | gcaacagttactgcgacgtg | 205–244 | 234 |
| 16E6-2R-inner | ggacacagtggcttttgaca | 419–438 |  |
| 16E6-2F-outer | gcaacagttactgcgacgtg | 205–224 | 386 |
| 16E6-3R-outer | tcatgcaatgtaggtgtatctcc | 568–590 |  |
| 16E6-3F-inner | cagcaatacaacaaaccgttg | 371–391 | 220 |
| 16E6-3R-inner | tcatgcaatgtaggtgtatctcc | 568–590 |  |
| 16LCR-1F-outer | gaaaacgaaaagctacaccca | 7084–7104 | 497 |
| 16LCR-2R-outer | gtgcaggtcaggaaaacag | 7562–7580 |  |
| 16LCR-1F-inner | gaaaacgaaaagctacaccca | 7084–7104 | 285 |
| 16LCR-1R-inner | caatgaataaccacaacacaatta | 7345–7368 |  |
| 16LCR-2F-inner | gcttgtgtaactattgtgtcatg | 7289–7311 | 292 |
| 16LCR-2R- inner | gtgcaggtcaggaaaacag | 7562–7580 |  |
| 16LCR-3F-outer | acttgtacgtttcctgcttg | 7525–7544 | 483 |
| 16LCR-4R-outer | tgcagttctcttttggtgc | 85–103 |  |
| 16LCR-3F-inner | acttgtacgtttcctgcttg | 7525–7544 | 350 |
| 16LCR-3R-inner | gtgtaacccaaaatcggtttgc | 7853–7874 |  |
| 16LCR-4F-inner | gtcaccctagttcatacatga | 7777–7797 | 231 |
| 16LCR-4R-inner | tgcagttctcttttggtgc | 85–103 |  |

**Table 2**: PCR protocols

1. First PCR (Outer primers)

The amplification of the 4 outer regions (16E6 1F-2R, 16E6 2F-3R, 16LCR 1F-2R, 16LCR 3F-4R) was performed with the AmpliTaq DNA Pol (Applied Biosystems^TM^) and according to the following protocol :

|  | **Concentration** | **1x (µl)** | **PCR program** |  |  | **Cycles** |
| --- | --- | --- | --- | --- | --- | --- |
| 10X Buffer | 1X | 2.5 |  |  |  |  |
| 10 mM dNTP Mix | 0.2 mM each | 0.5 | Holding stage | 2 min | 95⁰C | 1x |
| 25 mM MgCl2 (with Buffer II) | 1.5 mM | 1.5 |  |  |  |  |
| 10 uM forward primer | 0.2 µM | 0.5 | Cycling stage | 15 sec | 95⁰C | 45x |
| 10 uM reverse primer | 0.2 µM | 0.5 |  | 30 sec | 55⁰C |  |
| AmpliTaq DNA Pol (5U/uL) | 1.25 / 50 µL rxn | 0.13 |  | 45 sec | 72⁰C |  |
| Nuclease-free water | - | 16.8 |  |  |  |  |
| DNA sample |  | 2.5 | Final extension | 5 min | 72⁰C |  |
| **Total** |  | 25 |  | forever | 8⁰C | 1x |

1. Nested PCR (Inner primers)

The amplification of the 7 inner regions (16E6 1F-1R, 16E6 2F-2R, 16E6 3F-3R, 16LCR 1F-1R, 16LCR 2F-2R, 16LCR 3F-3R, 16LCR 4F-4R) was performed with the AmpliTaq DNA Pol (Applied Biosystems^TM^) and according to the following protocol :

|  | **Concentration** | **1x (µl)** | **PCR program** |  |  | **Cycles** |
| --- | --- | --- | --- | --- | --- | --- |
| 10X Buffer | 1X | 2.5 |  |  |  |  |
| 10 mM dNTP Mix | 0.2 mM each | 0.5 | Holding stage | 2 min | 95⁰C | 1x |
| 25 mM MgCl2 (with Buffer II) | 1.5 mM | 1.5 |  |  |  |  |
| 10 uM forward primer | 0.2 µM | 0.5 | Cycling stage | 15 sec | 95⁰C | 45x |
| 10 uM reverse primer | 0.2 µM | 0.5 |  | 30 sec | 55⁰C |  |
| AmpliTaq DNA Pol (5U/uL) | 1.25 / 50 µL rxn | 0.13 |  | 30 sec | 72⁰C |  |
| Nuclease-free water | - | 18.4 |  |  |  |  |
| PCR product (first PCR) |  | 1 | Final extension | 5 min | 72⁰C |  |
| **Total** |  | 25 |  | forever | 8⁰C | 1x |

**Amplification of modified Fast Aneuploidy Screening Test-Sequencing System (mFAST-Seq)**

**Table 3:** Primers sequences

|  | **Names** | **Sequences (5′ - 3′)** |
| --- | --- | --- |
| **LINE-1 PCR** | LINE1_F | tctttccctacacgacgctcttccgatctacacagggaggggaacat |
|  | LINE1_R | gtgactggagttcagacgtgtgctcttccgatcttgccatggtggtttgct |
|  |  |  |
| **Index PCR** | Index_Forward | aatgatacggcgaccaccgagatctacactctttccctacacgacgctcttccgatct |
|  | Rev_Index1 | caagcagaagacggcatacgagatcgtgatgtgactggagttcagacgtgtgctcttccgatct |
|  | Rev_Index2 | caagcagaagacggcatacgagatacatcggtgactggagttcagacgtgtgctcttccgatct |
|  | Rev_Index3 | caagcagaagacggcatacgagatgcctaagtgactggagttcagacgtgtgctcttccgatct |
|  | Rev_Index4 | caagcagaagacggcatacgagattggtcagtgactggagttcagacgtgtgctcttccgatct |
|  | Rev_Index5 | caagcagaagacggcatacgagatcactgtgtgactggagttcagacgtgtgctcttccgatct |
|  | Rev_Index6 | caagcagaagacggcatacgagatattggcgtgactggagttcagacgtgtgctcttccgatct |
|  | Rev_Index7 | caagcagaagacggcatacgagatgatctggtgactggagttcagacgtgtgctcttccgatct |
|  | Rev_Index8 | caagcagaagacggcatacgagattcaagtgtgactggagttcagacgtgtgctcttccgatct |
|  | Rev_Index9 | caagcagaagacggcatacgagatctgatcgtgactggagttcagacgtgtgctcttccgatct |
|  | Rev_Index10 | caagcagaagacggcatacgagataagctagtgactggagttcagacgtgtgctcttccgatct |
|  | Rev_Index11 | caagcagaagacggcatacgagatgtagccgtgactggagttcagacgtgtgctcttccgatct |
|  | Rev_Index12 | caagcagaagacggcatacgagattacaaggtgactggagttcagacgtgtgctcttccgatct |
|  | Rev_Index13 | caagcagaagacggcatacgagatttgactgtgactggagttcagacgtgtgctcttccgatct |
|  | Rev_Index14 | caagcagaagacggcatacgagatggaactgtgactggagttcagacgtgtgctcttccgatct |
|  | Rev_Index15 | caagcagaagacggcatacgagattgacatgtgactggagttcagacgtgtgctcttccgatct |
|  | Rev_Index16 | caagcagaagacggcatacgagatggacgggtgactggagttcagacgtgtgctcttccgatct |
|  | Rev_Index18 | caagcagaagacggcatacgagatgcggacgtgactggagttcagacgtgtgctcttccgatct |
|  | Rev_Index19 | caagcagaagacggcatacgagattttcacgtgactggagttcagacgtgtgctcttccgatct |
|  | Rev_Index20 | caagcagaagacggcatacgagatggccacgtgactggagttcagacgtgtgctcttccgatct |
|  | Rev_Index21 | caagcagaagacggcatacgagatcgaaacgtgactggagttcagacgtgtgctcttccgatct |
|  | Rev_Index22 | caagcagaagacggcatacgagatcgtacggtgactggagttcagacgtgtgctcttccgatct |
|  | Rev_Index23 | caagcagaagacggcatacgagatccactcgtgactggagttcagacgtgtgctcttccgatct |
|  | Rev_Index25 | caagcagaagacggcatacgagatatcagtgtgactggagttcagacgtgtgctcttccgatct |
|  | Rev_Index27 | caagcagaagacggcatacgagataggaatgtgactggagttcagacgtgtgctcttccgatct |

Nucleotides highlighted in red correspond to the specific Index sequence which differentiate each primer.

**Table 4**: PCR protocols

1. LINE-1 PCR

After dilution of all DNA sample at 1 ng/ µl, amplification of LINE-1 region was performed with the Phusion Hot Start II DNA pol (Thermo Fisher Scientific, ref : F549L) and according to the following protocol :

|  | **Concentration** | **1x (µl)** | **PCR program** |  |  | **Cycles** |
| --- | --- | --- | --- | --- | --- | --- |
| 5x Phusion HF buffer | - | 10 |  |  |  |  |
| LINE1_F primer | 10 µM | 1.25 | Holding stage | 2 min | 98⁰C | 1x |
| LINE1_R primer | 10 µM | 1.25 |  |  |  |  |
| dNTP's | 10 mM | 1 | Cycling stage | 10 sec | 98⁰C | 8x |
| Phusion Polymerase | - | 1 |  | 2 min | 57⁰C |  |
| Nuclease-free water | - | 25.5 |  | 2 min | 72⁰C |  |
| DNA sample | 1 ng/µl | 10 |  |  |  |  |
| **Total** |  | 50 |  | forever | 8°C | 1x |

PCR products were cleaned with 70 µl of AMPure Beads (Beckman Coulter, Brea, CA, USA) and re-suspended in 10ul of 1x TE-buffer.

1. INDEX PCR

PCR products from LINE-1 PCR were used for the Index PCR using the Phusion Hot Start II DNA pol (Thermo Fisher Scientific, ref : F549L) and according to the following protocol :

|  | **Concentration** | **1x (µl)** | **PCR program** |  |  | **Cycles** |
| --- | --- | --- | --- | --- | --- | --- |
| 5x Phusion HF buffer | - | 10 |  |  |  |  |
| Index_Forward | 10 µM | 1.25 | Holding stage | 2 min | 98⁰C | 1x |
|  |  |  |  |  |  |  |
| dNTP's | 10 mM | 1 | Cycling stage | 10 sec | 98⁰C | 18x |
| Phusion Polymerase | - | 1 |  | 15 sec | 65⁰C |  |
| Nuclease-free water | - | 25.5 |  | 15 sec | 72⁰C |  |
| PCR product (LINE-1 PCR) | - | 10 |  |  |  |  |
| **Total** |  | 50 |  | forever | 8°C | 1x |

Sample-specific primers were added separately from the mix PCR in each well of the plate (1.25 µl of sample-specific primer at 10 µM).

A second cleaning step using 70 µl of AMPure Beads (Beckman Coulter, Brea, CA, USA) was performed and DNA were re-suspended in 10 µl of 1x TE-buffer.

Finally, the qualification and quantification of the PCR products were performed on an Agilent Bioanalyzer using a 7500 DNA kit (Agilent, Santa Clara, USA) and L1 amplicon libraries were pooled equimolarly and sequenced on an Illumina iSeq 100 generating 150 bp single reads.
